# Effect of altitude and solvent on *Psidium guajava* Linn. leaves extracts: phytochemical analysis, antioxidant, cytotoxicity and antimicrobial activity against food spoilage microbes

**DOI:** 10.1186/s13065-023-00948-9

**Published:** 2023-04-13

**Authors:** Rita Majhi, Rukusha Maharjan, Mitesh Shrestha, Aatish Mali, Angisha Basnet, Manish Baral, Rabin Duwal, Rojlina Manandhar, Prajwal Rajbhandari

**Affiliations:** 1 Department of Natural Product and Green Chemistry, Research Institute for Bioscience and Biotechnology (RIBB), Kathmandu, Nepal; 2 Department of Plant Physiology and Environmental Sciences, Research Institute for Bioscience and Biotechnology (RIBB), Kathmandu, Nepal; 3 Department of Applied Microbiology and Food Technology, Research Institute for Bioscience and Biotechnology (RIBB), Kathmandu, Nepal

**Keywords:** Guava leaves, Altitudinal effect, Solvent effect, Antioxidant, Antimicrobial, Food loss, Natural preservatives

## Abstract

**Background:**

Guava (*Psidium guajava* Linn.) has been traditionally used in the treatment of a wide range of diseases due to its rich content of secondary metabolites.

**Aim:**

This study was aimed to evaluate the effect of altitude and solvent systems on guava leaves crude extract’s phenolics and flavonoid content, antioxidant, antimicrobial, and toxicity nature.

**Methods:**

Guava leaves were collected from three different geographical locations in Nepal while solvents with an increasing polarity index were used for extraction. The yield percentage of extracts was calculated. Total Phenolic Content, Total Flavonoid Content, and antioxidant activity were determined by the Folin-Ciocalteu method, Aluminium chloride colorimetric method, and DPPH (2,2′-Diphenyl-1-picrylhydrazyl) assay respectively. The quantification of fisetin and quercetin was performed using the HPLC with method validation. The antimicrobial activity of the extracts was tested against bacteria and fungus isolated from spoiled fruits and vegetables and identified through 16s and 18s rRNA sequencing. Finally, Brine Shrimp Lethality Assay (BSLA) was used for testing the toxicity of the extracts.

**Results:**

The phenolic and total flavonoid content was found to be higher in ethanol extract (331.84 mg GAE/g dry extract) and methanol extract (95.53 mg QE/g dry extract) from Kuleshwor respectively. Water extract of guava leaves from Kuleshwor (WGK) did not show significantly different antioxidant activity when compared to methanol and ethanol extracts. Fisetin and quercetin were higher in WGK (1.176 mg/100 g) and (10.967 mg/100 g) dry extract weight respectively. Antibacterial activity against food spoilage bacteria was dose-dependent and found to be highest for all the extracts from different solvents and altitudes at higher concentrations (80 mg/ml). Similarly, methanol and ethanol guava extracts from all locations showed antifungal activity against *Geotrichum candidum* RIBB-SCM43 and *Geotrichum candidum* RIBB-SCM44. WGK was found to be non-toxic.

**Conclusion:**

Our study concludes that the antioxidant and antimicrobial activity of WGK was found to be similar statistically to that of methanol and ethanol extracts of Bishnupur Katti and Mahajidiya. These results suggest the possibility of using water as a sustainable solvent to extract natural antioxidant and antimicrobial compounds which can further be used as natural preservatives to extend the shelf life of fruits and vegetables.

**Supplementary Information:**

The online version contains supplementary material available at 10.1186/s13065-023-00948-9.

## Introduction

*Psidium guajava* Linn*.*, commonly known as guava, is a tropical tree from the Myrtaceae family. It is widely distributed across the tropical and subtropical regions of the world and it has sweet and aromatic fruits [1]. In Nepal, guava is the second most important fruit following orange encompassing a cultivation range of 115 to 1600 m [2]. This fruit crop could be grown from sea level to an altitude of 1515 m above sea level [3]. Different parts of the plant are widely used as food and in traditional medicine around the world to treat a wide range of diseases like gastroenteritis, dysentery, stomach pain, and wounds [4]. Guava leaves consist of a large number of essential oils, polysaccharides, minerals, enzymes, alkaloids, steroids, glycosides, tannins, flavonoids, and saponins which are known for their high antioxidant, antibacterial and anti-diabetic properties [5, 6].

Fruits and vegetables are an essential supply of nutrition. However, essential compounds such as vitamins, and minerals could be lost during harvest, storage, etc. leading to nutritional degradation and spoilage rendering them undesirable for consumption. In 2019, the Food and Agriculture Organization (FAO) estimated that 14% of food valued at an estimated 400 billion USD was lost between harvest and distribution globally [7]. In Nepal, 20–50% of food loss is due to spoilage and infestation, and 15–40% is lost during post-harvest [8]. Physical damage, microbial contamination, and physiological activity are the major causes of food loss [9]. Among microbes, bacteria and fungi constitute the major food spoilage microorganisms. To mitigate these losses, farmers and food industries use chemical preservatives such as sodium benzoate, sodium nitrate, and sulfur dioxide to extend the shelf-life of fruits and vegetables; however, these chemicals can have harmful effects on human health [10]. Hence, there is increasing consumer interest in natural preservatives. Extracts from different plant parts exhibit high antimicrobial, antioxidant activity, and are generally recognized as safe (GRAS) [9, 11]. Natural preservatives derived from plant and marine sources have been shown to possess a higher ability to increase the quality and shelf-life of the produce [12, 13].

Altitude is one of the factors that play a major role in the biosynthesis of secondary metabolites in plants. Elevational gradients have been shown to alter the content and composition of secondary metabolites like phenolics, flavonoids, terpenoids, alkaloids, and essential oils that further determine their free radical scavenging capacity [14–16]. Along with altitude, solvent also performs a major role in the extraction of different bioactive compounds from plant materials. Different compounds are extracted from different solvent systems with varying polarity indexes [17]. Organic solvents like methanol, ethanol, acetone, hexane, and ethyl acetate have been used for extracting phenolic compounds from different plant parts for ages [18]. The polarity of the solvent in conjunction with the characteristics of bioactive compounds mainly determines the activity potential of the extract. Owing to the toxic nature of organic solvents like methanol, green solvents like water are being opted for extraction. Additionally, cost-effectiveness has also made water an excellent candidate for extraction. Therefore, this study aimed to compare the phytochemicals, antioxidants, and antimicrobial potential of guava leaves extracts extracted on different solvents from varying altitudes of Nepal. Furthermore, the toxicity of the extracts was also assessed to explore their potential as natural preservative-based edible coating systems.

## Materials and methods

### Collection and identification of plant materials

**Fig. 1:**
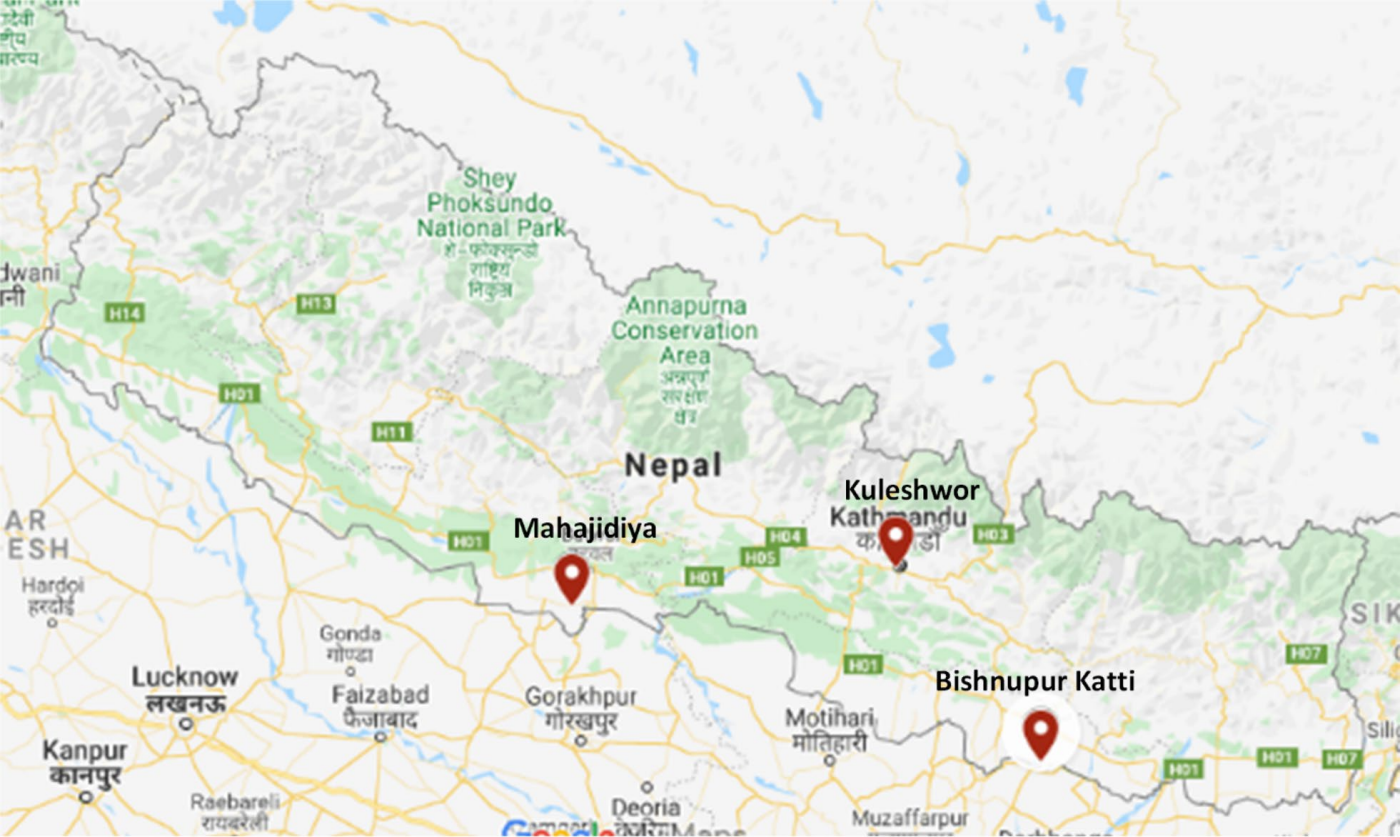
*Psidium guajava* Linn. leaves collections from three different locations in Nepal

Fresh guava leaves were collected in June–August from three locations in Nepal with different altitudes viz. Kuleshwor (Kathmandu—1306 m, 27.6900°N, 85.2955°E), Bishnupur Katti (Siraha—80 m, 26.8229°N, 86.4390°E), and Mahajidiya (Rupandehi—103 m, 27.6264°N, 83.3789°E) (Fig. 1). Plant materials were identified and deposited at the National Herbarium and Plant Laboratory, Godawari, Lalitpur, Nepal. The voucher codes for Kuleshwor, Bishnupur Katti, and Mahajidiya were *Rojlina Manandhar* KG1 (KATH), *Rojlina Manandhar* BG2 (KATH), and *Rojlina Manandhar* MG3 (KATH) respectively.

### Extracts preparation

Freshly collected leaves were washed with tap water to remove any dust particles and shade-dried for 2–3 weeks at room temperature before grinding to a fine powder. The powder (15 g) was weighed and placed in a 250 ml conical flask containing each 150 ml methanol (100%), ethanol (75%), and distilled water (D/W) in a 1:10 ratio. The mixture was then kept at room temperature for 48 h followed by filtration using Whatman filter paper (15 µm pore size). The filtrate was stored at 4 °C while residue was re-soaked in 75 ml of respective solvent and kept overnight at room temperature. This mixture was then kept in a water bath at 42 °C for 3 h followed by filtration. The second extract was mixed with the first extract and concentrated by evaporating in a rotary evaporator (EYELA, Japan) under reduced pressure and temperature at 40 °C with slight modification [19, 20]. Crude extracts were kept at 4 °C until further analysis. All experiments were performed in triplicates. All of the extracts were labeled as Solvent, Guava, and Location i.e. methanolic extract of guava from Kuleshwor was coded as MGK and likewise for other extracts (Table 1).

**Table 1:** Codes for *Psidium guajava* L*.*, leaves extracts with different solvents from varying altitudes

S.n.	Name of the solvent	Location	Sample code
1	Methanol	Kuleshwor	MGK
2		Bishnupur Katti	MGB
3		Mahajidiya	MGM
4	Ethanol	Kuleshwor	EGK
5		Bishnupur Katti	EGB
6		Mahajidiya	EGM
7	Water	Kuleshwor	WGK
8		Bishnupur Katti	WGB
9		Mahajidiya	WGM

### Phytochemical analysis

#### Determination of total phenolic content

The total phenolic content (TPC) of the extracts was determined by the modified Folin-Ciocalteu reagent (FCR) assay [21, 22]. Briefly, 20 µl (1 mg/ml) of the extract was kept in 96 well plates along with 100 µl FC reagent (1:10 dilution in D/W). The mixture was kept for 5 min at room temperature in dark conditions. Then, 80 µl (7.5% in D/W) of sodium carbonate (Na_2_Co_3_) was added to the mixture. The plates were then incubated for 2 h in dark conditions and the absorbance was measured at 765 nm using a microplate reader (Bio Tek, USA). Gallic acid was used as standard, and the results were expressed as mg gallic acid equivalent/gram (mg GAE/g dry extract).

#### Determination of total flavonoid content

The total flavonoid content (TFC) of extracts was determined by using the colorimetric Aluminium chloride (AlCl_3_) method [22, 23]. Briefly, 2% AlCl_3_ in methanol was prepared. Then, 100 µl of each of the extracts were mixed with AlCl_3_ solution (100 µl), kept at room temperature for 10 min, followed by centrifugation (NUVE, Turkey) at 2000 rpm for 4 min and the absorbance was measured at 415 nm. The calibration curve was prepared using various concentrations of quercetin in methanol. TFC results were expressed as mg quercetin equivalent/gram (mg QE/g dry extract).

### DPPH radical scavenging activity

In-vitro antioxidant potential of guava leaves was assessed by DPPH assay with slight modification [22, 24]. Different concentrations (5, 10, 20, 40, 80, 160, and 320 µg/ml) of plant extracts (50 µl) were mixed with 150 µl (0.1 mM) of DPPH (Sigma Aldrich, Germany) solution made in methanolin a 96-well plate. The plate was allowed to stand in a dark condition for 30 min and absorbance was taken at 520 nm using a microplate reader. Ascorbic acid was taken as a standard to calculate the antioxidant capacity and results were expressed in µg/ml. DPPH radical scavenging activity was calculated using the following formula:

$$\% {\text{ radical scavenging }} = \left[ {\left( {{\text{A}}_{0} - {\text{ A}}_{{1}} } \right)/{\text{A}}_{0} } \right] \, *{ 1}00$$

where, A_0_ = Absorbance of the control solution, A_1_ = Absorbance of extract/standard.

### HPLC analysis

#### Optimization of HPLC condition

The quantitative analysis of flavonoid standards—fisetin and quercetin (Sigma-Aldrich, USA) was optimized using a lab solution software and an HPLC system of LC2030 (Shimadzu, Japan) on the C18 column (Luna Omega 3 um Polar C18 100, 250 × 4.6 mm, Phenomenex, USA) with slight modification [25, 26]. The solvents used for mobile phase A and phase B were HPLC grade water (Thermo Fisher Scientific, India) with pH-2.15 and acetonitrile respectively. The solvents were degassed for 30 min using a sonicator (Faithful Instrument, China). The gradient elution condition was set at 5 min, 35% B; at 10 min, 40% B; at 15 min, 50% B; and stopped at 20 min. The flow rate was 0.5 ml/min with an autosampler injection volume of 20 µl. The column temperature was 40 °C, and the UV–visible detector was set at 340 nm.

#### Preparation of standard and sample solution

The stock solutions of fisetin and quercetin were prepared by dissolving in HPLC grade methanol at the concentration of 1 mg/ml. Both stock solutions were serially diluted up to 100 µg/ml, 50 µg/ml, 25 µg/ml, and 12.5 µg/ml to plot the standard calibration curve. 5 mg of WGK, WGB, and WGM sample was dissolved in HPLC grade water using a sonicator for 20 min and the solution was filtered by using a 0.2 µm filter (Sartorius Minisart filter, Germany) before injection [26].

### Method validation

The method was validated for linearity i.e. limit of detection and quantification (LOD and LOQ) following the analysis reports [26] and International Conference on Harmonization (ICH) guidelines [27].

### Quantification of guava leaves water extract

The new validated analytical method was applied for the simultaneous determination of fisetin and quercetin in different samples of guava leaves water extract. The quantification of both fisetin and quercetin was done by linear regression of the standards. Each sample was performed in triplicates [28].

### Microbiological analysis

#### Isolation of food spoilage microorganisms

Spoiled samples of fruits and vegetables (Banana, Papaya, Apple, Mango, Brinjal, Tomato, and Orange) were collected from three locations: Balkhu, Kalimati, and Dhulikhel since they are the major fruits and vegetable collection centers while Dhulikhel is a major eastern access point to Kathmandu. 1 g of each sample was smashed in a mortar and serially diluted. The dilution was spread on Nutrient Agar (HiMedia Laboratory, India) for bacteria and Potato Dextrose Agar (HiMedia Laboratory, India) for fungus isolation. The plates were incubated at 37 °C and 28 °C respectively. Pure colonies were identified through biochemical and molecular characterization methods. Glycerol stocks of the isolated cultures were prepared and stored at − 20 °C.

#### Polymerase chain reaction (PCR) and sequence analysis

Bacteria and fungi were identified through 16s and 18s rRNA sequencing. PCR was performed using universal primers of 16s and 18s rRNA (Table 2). Confirmed PCR products were sent to Genotech Inc., South Korea for sequencing. The sequences were assembled in MEGA software version X and identified using BLAST. All the generated sequences have been deposited in the NCBI (National Center for Biotechnology Information) database.

**Table 2:** PCR conditions of 16 s and 18 s rRNA

Gene	Initial denaturation	Denaturation	Annealing	Extension	Final extension	Hold
16s rRNA	95 °C	95 °C	54 °C	72 °C	72 °C	4 °C
	2 min	30 s	30 s	160 s	5 min	
			29 cycles			
18s rRNA	95 °C	95 °C	50 °C	72 °C	72 °C	4 °C
	2 min	30 s	30 s	70 s	5 min	
			29 cycles			

#### Antibacterial assay of guava leaves extract against isolated food spoilage microorganisms

Antibacterial activity of guava leaves extracts was carried out using the filter paper disc method on Mueller Hinton Agar (MHA) (HiMedia Laboratory, India) plates. The bacterial isolates were adjusted to 0.5 McFarland standard solutions. The bacterial inoculum was uniformly spread using sterile cotton swabs on MHA plates. Then, 6 mm diameter filter paper discs impregnated with 20 µl aliquots of different concentrations of extracts (40 mg/ml, 60 mg/ml, and 80 mg/ml) were kept onto the plates. For each bacterial isolate, methanol, ethanol, and water was used as negative control while Chloramphenicol (30 mcg) was taken as a positive control. The zone of inhibition was measured after the plates were incubated at 37 °C for 16–18 h [29]. The minimum inhibitory concentration (MIC) and minimum bactericidal concentration (MBC) of WGK were determined by using broth micro-dilution assay [30].

### Antifungal assay

Fungal isolates were cultured in Potato Dextrose Broth (PDB) (HiMedia Laboratory, India) in a shaking incubator at 28 °C for 3 days. The fungal inoculum was uniformly spread using sterile cotton swabs on MHA plates. Then, 6 mm diameter filter paper discs impregnated with 20 µl aliquots of different concentrations of extracts (40 mg/ml, 60 mg/ml, and 80 mg/ml) were placed on the plates. For each fungal isolate, methanol, ethanol, and water was used as a negative control. The plates were then incubated at 28 °C for 2–3 days and the zone of inhibition was measured [31].

### Cytotoxicity assay

In vitro toxicity of guava leaves, extracts were performed by brine shrimp lethality assay [32]. It is a simple, rapid, and cheap assay used to test the cytotoxic potential of plant extracts. Brine shrimp (*Artemia salina*) eggs were hatched using artificial seawater (38 g/l NaCl, pH-8.3) in an incubator at 28 °C for 24 h in presence of fluorescence light. Different concentrations (1000, 500, 250, 125, 62.25, 31.25, and 15.56 µg/ml) of guava leaves extracts were prepared with seawater for toxicity tests. An equal volume (2.5 ml) of each concentration of the extract was added to 2.5 ml of seawater in each vial containing 10 active *A. salina* nauplii. Potassium Dichromate dissolved in seawater was used as a positive control. The effect of test samples was monitored after 24 h of incubation by counting the remaining live nauplii. Each test was performed in triplicates. LC_50_ (Median Lethal Concentration) values were obtained using probit regression analysis and compared with Meyer’s toxicity criteria, whereby extract with LC_50_ < 1000 µg/ml is considered toxic and LC_50_ > 1000 µg/ml as non-toxic [33].

### Statistical analysis

All experiments were performed in triplicates. Results of the replicates were expressed as mean ± standard deviation (SD). Statistical analysis was performed using SAS software (version 9.1) with analysis of variance (One-Way ANOVA). Experimental results were further analyzed for the Pearson correlation coefficient (R-square) between TPC, TFC, IC_50_, and yield percentage. A P-value of < 0.05 was taken as a significant difference.

## Results

### Determination of extraction yield

The yield percentage of dried crude guava extracts was calculated to the initial weight of powder taken for extraction (Table 3). It was observed that ethanol extract (21.48 ± 0.86%) from Kuleshwor and methanol extract (20.60 ± 0.74%) from Bishnupur Katti had a higher yield. Water extracts from all three locations had a significantly lower yield percentage compared to methanol and ethanol. MGK, MGM, and EGM extracts showed no significant difference in yield percentage. Similarly, MGB, EGK, and EGB extracts were also found to be non significantly different.

**Table 3:** Yield percentage of *Psidium guajava* Linn.leaves extracts collected from different locations along with solvents

Sample code	Yield percentage (%)
MGK	15.22 ± 0.17^a^
MGB	20.60 ± 0.74^b^
MGM	17.62 ± 2.08^a^
EGK	21.48 ± 0.86^b^
EGB	20.02 ± 1.39^b^
EGM	15.69 ± 3.24^a^
WGK	9.20 ± 0.66^d^
WGB	7.56 ± 0.62^d^
WGM	5.51 ± 1.37^e^

Data are presented as the mean ± SD (n = 3). Lowercase letters (a-e) indicated a significant difference (p ˂ 0.05)

### Free radical scavenging activity

**Fig. 2:**
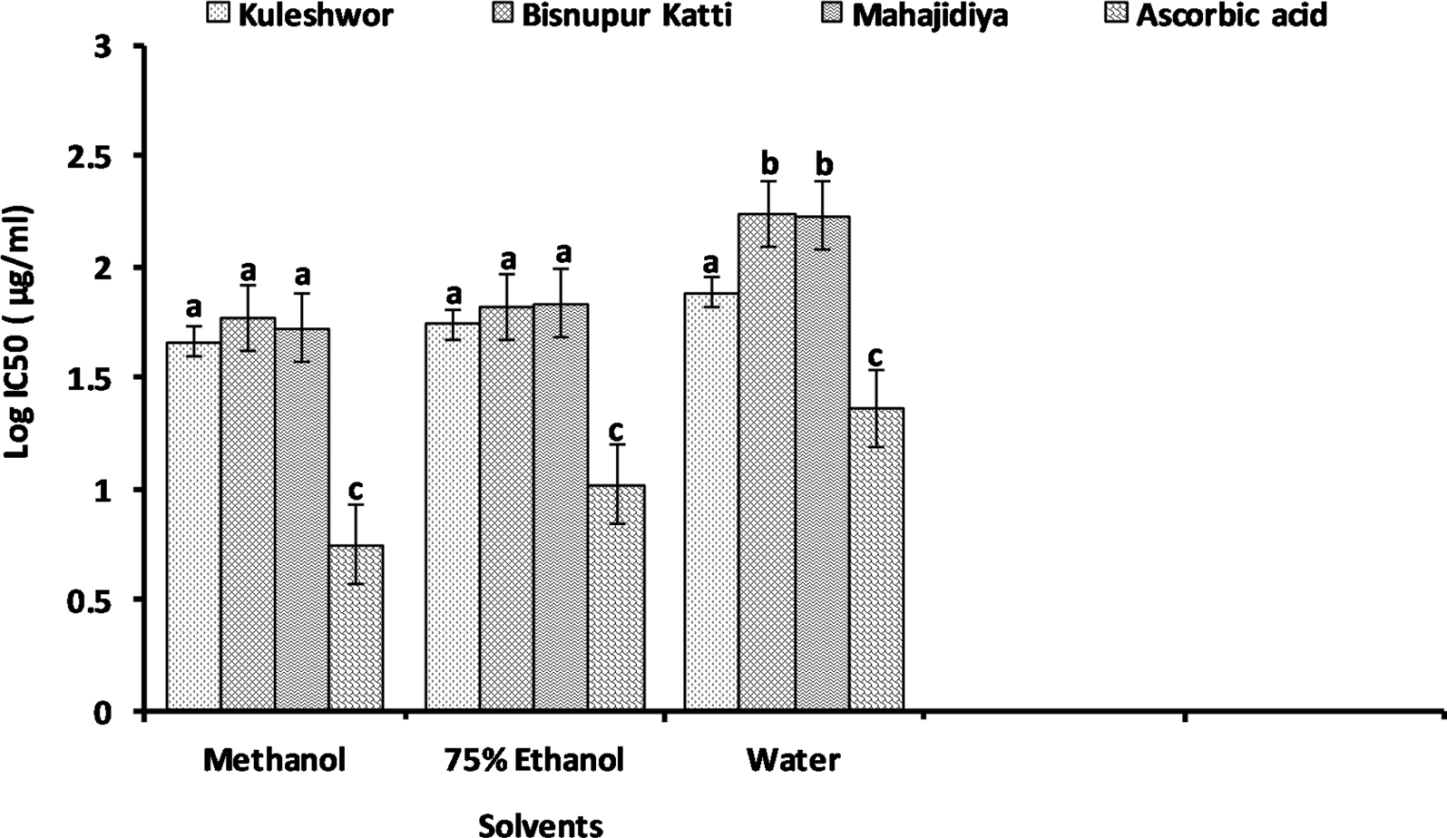
Scavenging ability of DPPH free radicals by *Psidium guajava* Linn. leaves extracts. Lowercase letters are significantly different (p < 0.05), and the data represent the mean of three replicate

The log IC_50_ value for WGK (1.882 µg/ml) was found to be comparable with the other two extracts (methanolic and ethanolic) from lower altitude locations (Fig. 2).

### Total phenol and flavonoid contents

**Fig. 3:**
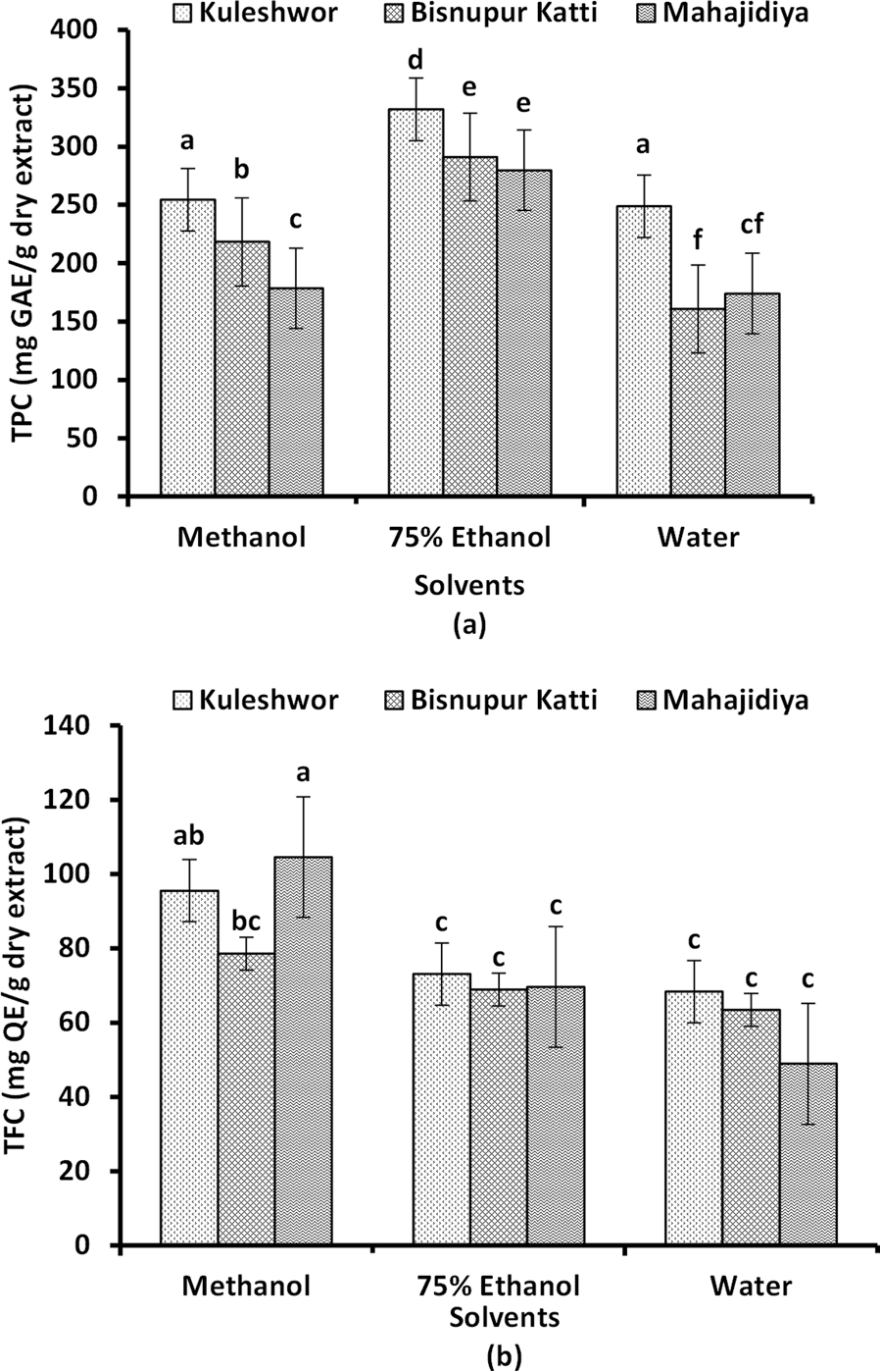
Phytochemical analysis of *Psidium guajava* Linn. leaves extracts with different solvents from different altitudes. **a** Total phenol content. **b** Total flavonoid content. Lowercase letters represent the level of significance (p < 0.05). GAE/g dry extract—gallic acid equivalent/gram, QE/g dry extract—quercetin equivalent/gram

Polyphenols are the major phytochemicals responsible for antioxidant properties. Ethanolic extracts showed the highest total phenolic content (331.84 mg GAE/g dry extract). Similarly, WGB and WGM had the lowest phenolic content but WGK had higher phenolic content (248.9 mg GAE/g dry extract) compared to MGK. In terms of altitude, Kuleshwor extracts had higher phenolic content in all three solvents (methanol, ethanol, and water) which shows altitude plays a role in the production of phenolic compounds in guava leaves. The highest flavonoid contents were observed in MGK (95.53 mg QE/g dry extract) and MGM (104.59 mg QE/g dry extract), while WGM (48.91 mg QE/g dry extract) had the lowest among all extracts. Ethanol and water extracts of all three locations did not show a significant difference in flavonoid content. The results of TPC and TFC are shown in (Fig. 3).

### Pearson’s correlation between IC_50_, TPC, TFC, and yield percentage

The relation between total phenolic, total flavonoid, antioxidant, and yield percentage is shown in (Table 4). IC_50_ showed negative correlation with yield percentage (R^2^ = − 0.71) as well as with TPC (R^2^ = − 0.55). However, TPC had a positive correlation with yield percentage (R^2^ = 0.6). Similar findings have been reported for *Garcinia lasoar* Pam [34].

**Table 4:** Pearson’s correlation between IC_50_, TPC, TFC, and Yield percentage

Parameters	IC_50_	TPC	TFC	Yield (%)
IC_50_	1	–	–	–
TPC	− 0.55	1	–	–
TFC	− 0.49	0.06	1	–
Yield	− 0.71	0.60	0.40	1

A significant difference (p < 0.05)

### HPLC chromatogram

**Fig. 4:**
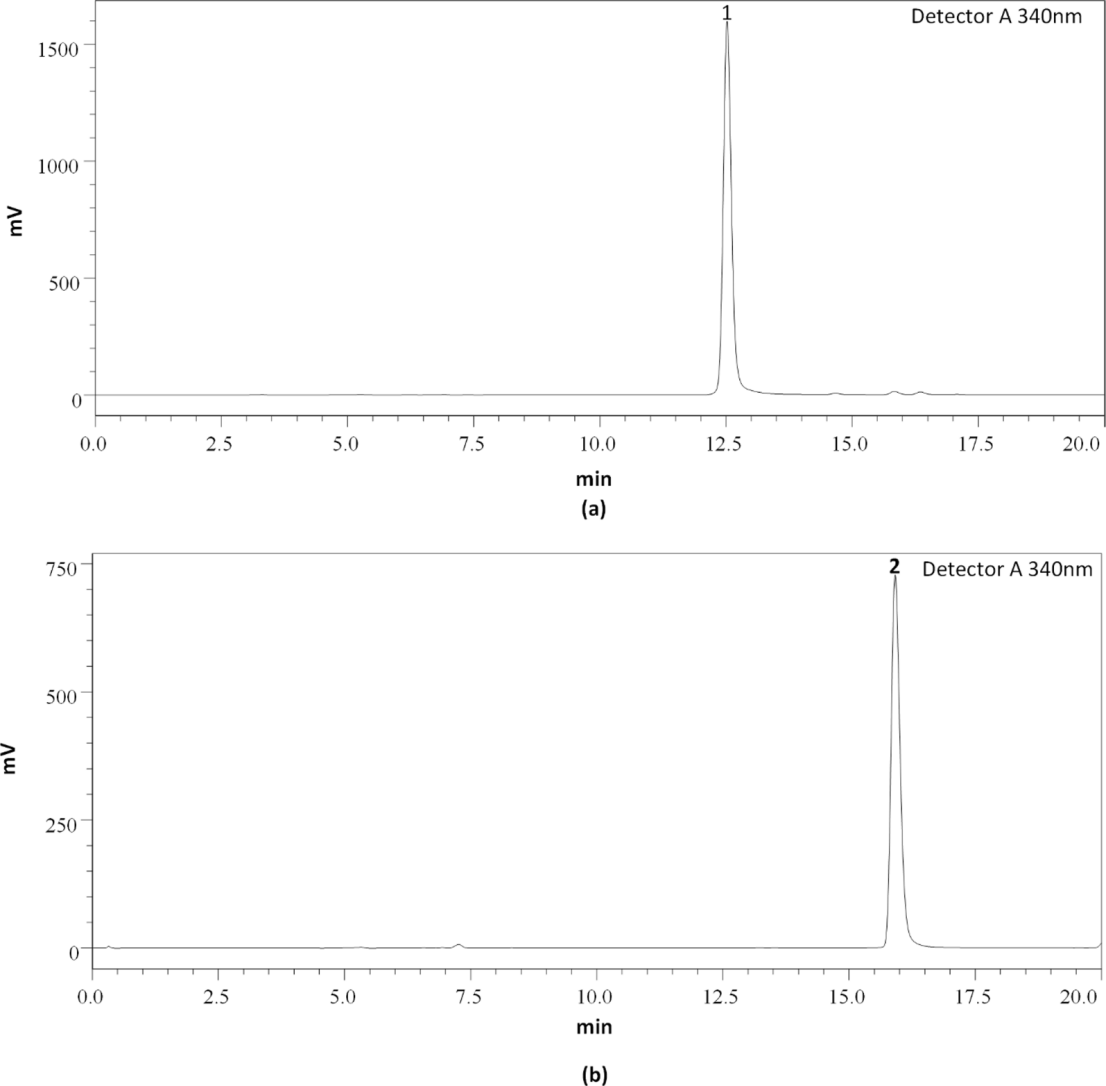
Chromatogram of Standard flavonoids **a** Fisetin and **b** Quercetin

**Fig. 5:**
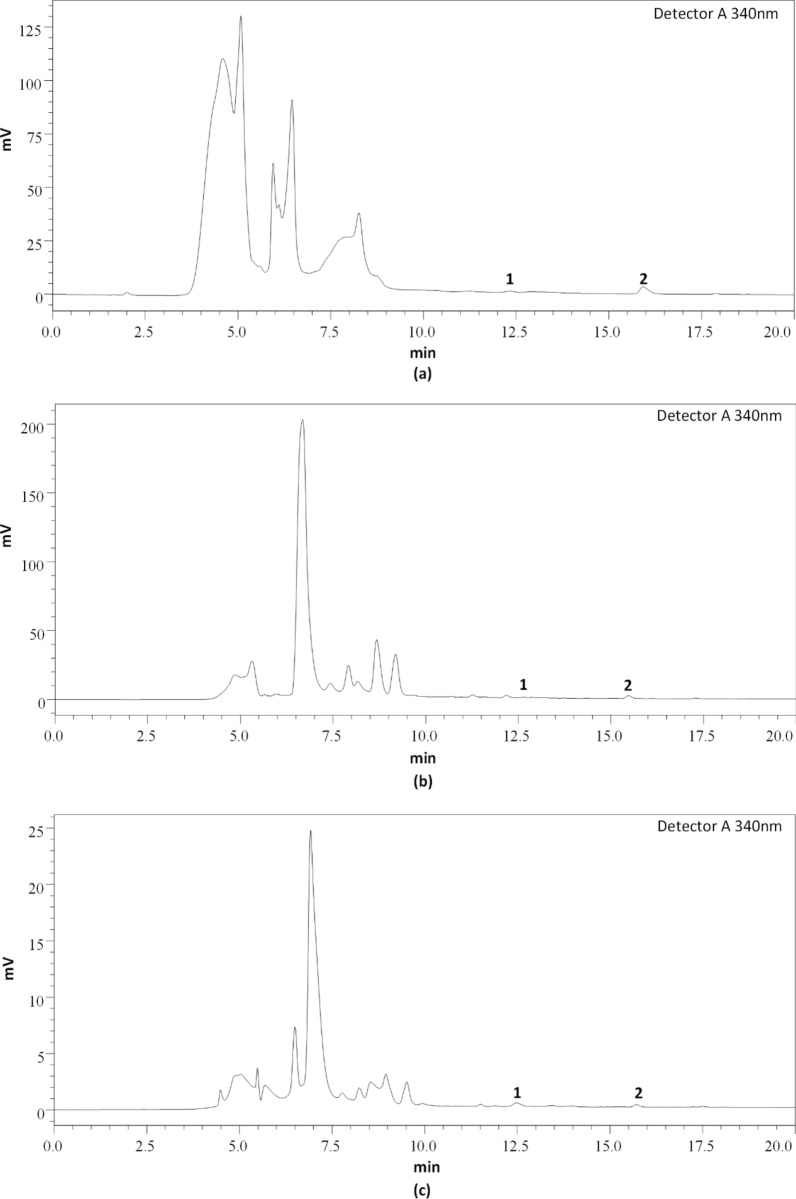
Chromatogram of *Psidium guajava* Linn. leaves extracts **a** WGK, **b** WGB, and **c** WGM; **1:** Fisetin and **2:** Quercetin

After the optimization of HPLC conditions, mobile phase A (HPLC grade water) and phase B (acetonitrile) were established with a detection wavelength of 340 nm for better chromatographic resolution. A column temperature of 40 °C with a flow rate of 0.5 mL/min with an analysis time of 20 min was optimized. The chromatogram of a standard mixture of fisetin and quercetin as well as the chromatogram of WGK, WGB, and WGM samples is represented in (Figs. 4 and 5).

### Method validation

The method validation of analysis was investigated for linearity to confirm the process used is selective. For linearity, four concentrations (100, 50, 25, and 12.5 µg/ml) for fisetin and quercetin were injected individually. The calibration curve equation was obtained by plotting the peak areas vs concentrations. The R-square values for fisetin and quercetin were 0.9998 and 0.9999 respectively, hence linearity was verified. The calibration curve, linear range, Limit of Detection (LOD), and Limit of Quantification (LOQ) are represented in (Table 5).

**Table 5:** LODs, LOQs, Linear range, and Regression equation of fisetin and quercetin

Standards	LOD (µg/ml)	LOQ (µg/ml)	Linear range (R^2^)	Regression equation
Fisetin	4.12	12.50	0.9998	y = 184397x − 215,817
Quercetin	1.97	5.99	0.9999	y = 87220x + 213,388

### Quantification of fisetin and quercetin in guava leaves water extracts

WGK, WGB, and WGM samples were quantitatively analyzed for the contents of fisetin and quercetin. Each sample was analyzed in triplicates. Total fisetin and quercetin contents were expressed as mg per 100 g dry extract. WGK had the highest (10.967 mg/100 g) of quercetin whereas the lowest quercetin was detected in WGB (4.478 mg/100 g) and WGM (0.792 mg/100 g) respectively. Similarly, WGK contained (1.173 mg/100 g), WGM (0.543 mg/100 g), and WGB (0.408 mg/100 g) of fisetin (Table 6).

**Table 6:** Quantification of fisetin and quercetin in *Psidium guajava* L. leaves extracts

Sample	Concentration (mg/100 g dry extract)	
	Fisetin	Quercetin
WGK	1.173 ± 0.09	10.967 ± 1.32
WGB	0.408 ± 0.06	4.478 ± 0.44
WGM	0.543 ± 0.05	0.792 ± 0.26

Data are presented as the mean ± SD (n = 3)

### Isolation and characterization of food spoilage microorganisms

A total of 34 bacteria and 7 fungi were isolated from different spoiled fruits and vegetables. All the 16s and 18s rRNA sequences have been deposited at National Center for Biotechnology Information (NCBI) with an accession number (Tables 7 and 8).

**Table 7:** Isolation and molecular characterization of bacteria from spoiled foods

Fruits and vegetables	Location	Bacterial sample (accession no.)	Identified as
Apple (*M. domestica*)	Balkhu (Kathmandu)—27.6863°N, 85.2949°E Kalimati (Kathmandu)—27.7000°N, 85.2891°E	RIBB-SCM1 (MK905700) RIBB-SCM2 (MK905701) RIBB-SCM3 (MK905702) RIBB-SCM4 (MK905703) RIBB-SCM5 (MK905705) RIBB-SCM6 (MK905706) RIBB-SCM7 (MK905707)	*Klebsiella* sp. *Klebsiella* sp. *Fictibacillus* sp. *Enterobacter* sp. *Bacillus* sp. *Bacillus* sp. *Methylorubrum* sp*.*
Orange *(C. sinensi)*	Dhulikhel (Kavrepalanchok)- 27.6253° N, 85.5561° E	RIBB-SCM21 (MK905720) RIBB-SCM22 (MK905721) RIBB-SCM23 (MK905722)	*Bacillus* sp*.* *Bacillus licheniformis* *Bacillus* sp.
Banana (*Musa*)	Balkhu (Kathmandu) Kalimati (Kathmandu)	RIBB-SCM8 (MK905707) RIBB-SCM9 (MK905708) RIBB-SCM10 (MK905709) RIBB-SCM11 (MK905710)	*Enterobacteriaceae bacterium* *Staphylococcus sciuri* *Bacillus* sp*.* *Pseudomonas* sp*.*
	Dhulikhel (Kavrepalanchok)	RIBB-SCM12 (MK905711) RIBB-SCM13 (MK905712) RIBB-SCM14 (MK905713) RIBB-SCM15 (MK905714) RIBB-SCM16 (MK905715)	*Pantoea dispersa* *Bacillus licheniformis* *Pantoea* sp. *Klebsiella pneumoniae* *Klebsiella* sp.
Mango (*M. indica*)	Balkhu (Kathmandu) Kalimati (Kathmandu)	RIBB-SCM19 (MK905718) RIBB-SCM20 (MK905719)	*Acinetobacterbaumannii* *Acinetobacterbaumannii*
Papaya (*C. papaya*)	Balkhu (Kathmandu) Kalimati (Kathmandu)	RIBB-SCM24 (MK905723) RIBB-SCM25 (MK905724) RIBB-SCM26 (MK905725) RIBB-SCM27 (MK905726)	*Enterobacter* sp. *Klebsiella* sp. *Staphylococcus* sp. *Bacillus* sp.
	Dhulikhel (Kavrepalanchok)	RIBB-SCM28 (MK905727) RIBB-SCM29 (MK905728) RIBB-SCM30 (MK905729) RIBB-SCM31 (MK905730) RIBB-SCM32 (MK905731)	*Enterobacter* sp. *Staphylococcus sciuri* *Klebsiella* sp. *Staphylococcus sciuri* *Staphylococcus sciuri*
Brinjal (*S. melongena*)	Dhulikhel (Kavrepalanchok)	RIBB-SCM17 (MK905716) RIBB-SCM18 (MK905717)	*Pseudomonas* sp*.* *Acinetobacter* sp.
Tomato (*S. lycopersicum*)	Dhulikhel (Kavrepalanchok)	RIBB-SCM33 (MK905732) RIBB-SCM34 (MK905733)	*Bacillus* sp. *Bacillus* sp.

Scientific name: *M. domestica* = *Malus domestica*, *C. sinensi* = *Citrus sinensis*, *M. indica* = *Mangifera indica*, *C. papaya* = *Carica papaya*, *S. melongena* = *Solanum melongena*, *S. lycopersicum* = *Solanum lycopersicum*

**Table 8:** Isolation and molecular characterization of fungi from spoiled foods

Fruits and vegetables	Location	Fungi sample (accession no.)	Identified as
Apple	Kalimati (Kathmandu)	RIBB-SCM35 (MK905741)	*Alternaria alternate*
Banana	Balkhu (Kathmandu)	RIBB-SCM38 (MK905744)	*Fusarium* sp.
		RIBB-SCM39 (MK905745)	*Fusarium* sp.
	Dhulikhel (Kavrepalanchok)	RIBB-SCM40 (MK905746)	*Fusarium* sp.
Papaya	Balkhu (Kathmandu)	RIBB-SCM41 (MK905747)	*Rhizopus oryzae*
Tomato	Dhulikhel (Kavrepalanchok)	RIBB-SCM43 (MK905749)	*Geotrichum candidum*
		RIBB-SCM44 (MK905750)	*Geotrichum candidum*

### Antibacterial and antifungal activity of guava leaves extracts

**Fig. 6:**
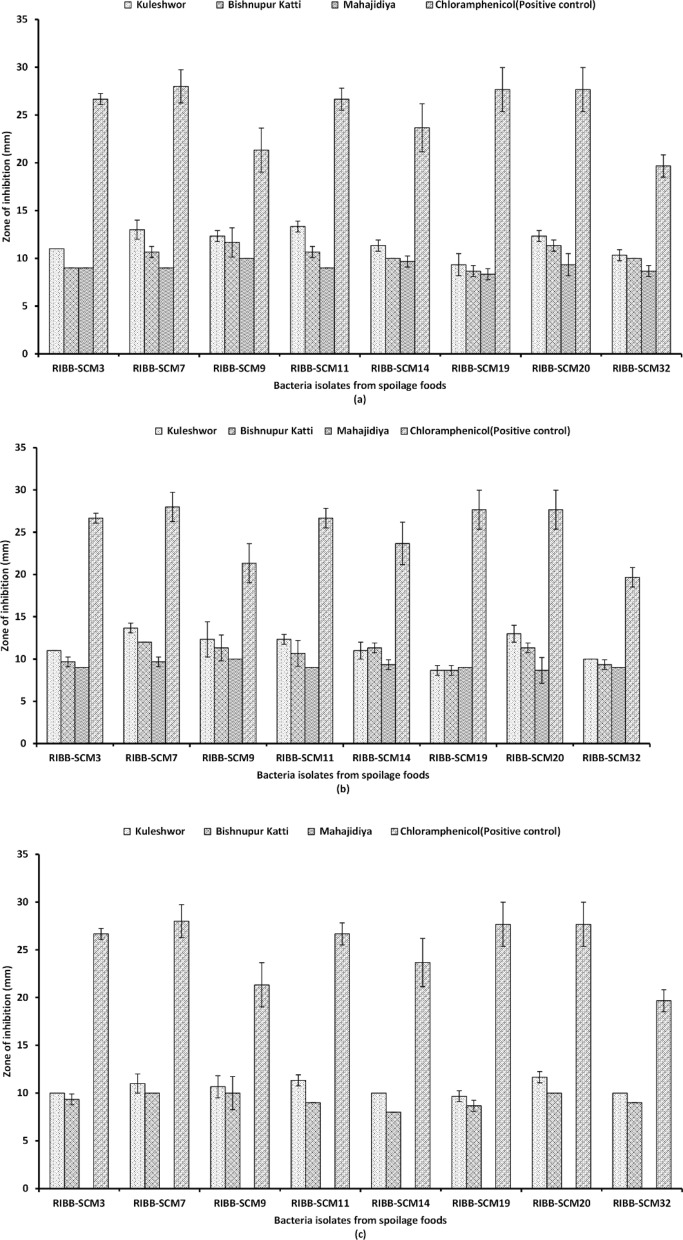
Antibacterial activity of *Psidium guajava* Linn. leaves extracts with different solvents of high concentration (80 mg/ml) from different locations measured in diameter of zone of inhibition (mm) against food spoilage bacteria. **a** Methanol extracts. **b** 75% ethanol extracts. **c** Water extracts. Each data represents the mean of three replicates

The presence of bioactive compounds or secondary metabolites corresponds with the antibacterial and antifungal activity of plant extracts [35]. Among 34 bacterial isolates MGK, EGK, and WGK showed the highest antibacterial activity at 80 mg/ml concentration against three Gram-positive bacteria (*Staphylococcus sciuri* RIBB-SCM9, *Fictibacillus* sp. RIBB-SCM3, *Staphylococcus sciuri* RIBB-SCM32) and five Gram-negative bacteria (*Methylorubrum* sp. RIBB-SCM7, *Acinetobacter baumannii* RIBB-SCM19, *Pseudomonas* sp*.* RIBB-SCM11*, Pantoea* sp. RIBB-SCM14, and* Acinetobacter baumannii* RIBB-SCM20) isolated from different spoiled tropical and temperate fruits like Banana, Papaya, Mango, Apple in comparison to extracts from lower altitude (Bishnupur Katti and Mahajidiya) (Fig. 6). The data also shows that WGM has no antibacterial activity in any of the concentrations used (Additional file 1: Table S1).

**Fig. 7:**
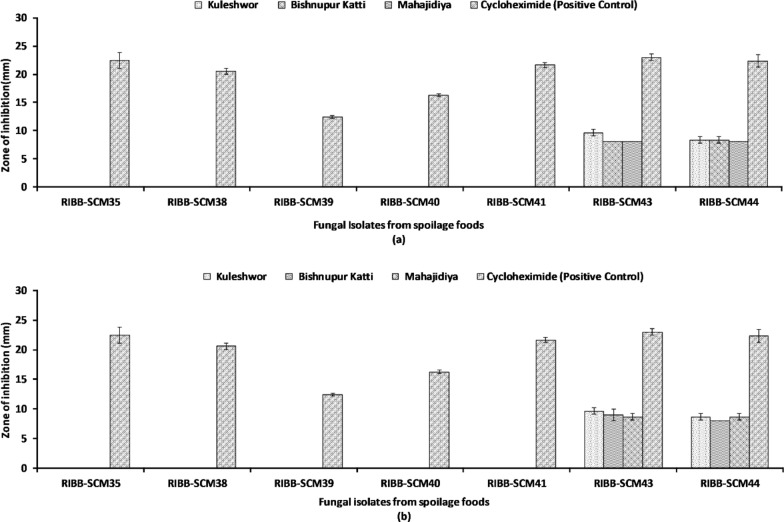
Antifungal activity of *Psidium guajava* Linn. leaves extracts with different solvents of high concentration (80 mg/ml) from different locations measured in diameter of zone of inhibition (mm) against fungi. **a** Methanol extracts. **b** 75% ethanol extracts. Each data represents the mean of three replicates

WGK had bactericidal effect against two Gram-positive bacteria (*Fictibacillus* sp. RIBB-SCM3 and *Staphylococcus sciuri* RIBB-SCM32) and two Gram-negative bacteria (*Methylorubrum* sp. RIBB-SCM7 and *Pseudomonas* sp*.* RIBB-SCM11) with a MIC of 7.81 mg/ml and MBC of 15.62 mg/ml (Table 9). Furthermore, only methanolic and ethanolic extracts from three different locations exhibited antifungal activity against fungal strains *Geotrichum candidum* RIBB-SCM43 and *Geotrichum candidum* RIBB-SCM44 isolated from Tomato (Fig. 7).

**Table 9:** Minimum inhibitory concentration and Minimum bactericidal concentration of WGK against bacterial isolates

Bacterial isolates	MIC concentration (mg/ml)	MBC concentration (mg/ml)	Streptomycin (mg/ml)
RIBB-SCM 3	7.81	15.62	0.97
RIBB-SCM 7	7.81	15.62	0.97
RIBB-SCM 9	–	–	–
RIBB-SCM 11	7.81	15.62	1.95
RIBB-SCM 14	–	–	–
RIBB-SCM 19	–	–	–
RIBB-SCM 20	–	–	–
RIBB-SCM 32	7.81	15.62	1.95

Streptomycin as a Positive Control

### Cytotoxicity assay of guava leaves extracts

LC_50_ value of methanol, ethanol and water extract of guava leaves was determined using probit regression analysis (Table 10). Water extracts from all three locations were found to be non-toxic, in which WGM had the highest LC_50_ value (5.6 × 10^4^ µg/ml) whereas methanol extract from all three locations and EGK were found to be toxic. In contrast, both EGB and EGM were found to be non-toxic.

**Table 10:** Cytotoxicity assay of *Psidium guajava* L., leaves extracts collected from different locations along with solvents and potassium dichromate represented by LC_50_, 95% CI, and correlation coefficient (R^2^)

Sample code	LC_50_ (µg/ml)	95% CI	Correlation coefficient (R^2^)
MGK	253.673 ± 47.18	136–370	0.8124
MGB	180.438 ± 33.51	97–263	0.9934
MGM	441.482 ± 185.07	− 18 to 901	0.9456
EGK	142.156 ± 15.35	104–180	0.8089
EGB	1538.75 ± 324.54	732–2344	0.9426
EGM	17,270.5 ± 21,352.00	− 35,770 to 70,311	0.8611
WGK	23,888.4 ± 5046.90	11,351–36,425	0.9396
WGB	6363.81 ± 1444.26	2776–9951	0.9318
WGM	56,008.1 ± 29,749.22	− 17,893 to 129,909	0.8764
Potassium dichromate (positive control)	16.15 ± 3.015	1	1

Lethality concentration (LC_50_) data are presented in the mean ± SD (n = 3)

95% CI = 95% Confidence interval

## Discussion

Plant extracts contain phytochemicals such as phenols, flavonoids, anthocyanin, and carotenoids that can donate electrons to free radicals and neutralize them. These compounds help plants to withstand stressful environmental conditions such as low temperature, high UV light impact, increased atmospheric pressure, length of vegetation period, etc. [36]. Yield and bioactive compounds extracted from plants are highly dependent on the properties of solvents [37]. Our findings have shown that the yield percentage of aqueous guava extracts is lower than methanol and ethanol extracts for all the locations. The complex formation between polyphenols and molecules such as protein, and carbohydrate are more extractable in methanol and ethanol than in water as they are better hydrogen donors and acceptors. Moreover, a good solvent system has high solvency and can cleave hydrogen bond as well. However, water being highly polar, is unable to cleave hydrogen and hydrophobic bonds between complex bioactive compounds, hence, the lower yield [37, 38]. Our result also shows EGK has higher yield percentage than MGK and WGK. It could be due to the percentage combination of organic and aqueous solvents (75% ethanol) that facilitates the extraction of chemicals that are extractable in both water and ethanol which highlights the role of water in polar alcohol solvents like ethanol for better extraction yield [39].

IC_50_ value and antioxidant properties of the plant extracts are inversely proportional. Our findings indicate that methanol and ethanol extracts have significantly lower IC_50_ values than water extracts. Interestingly, antioxidant activity of WGK is statistically similar to that of methanolic and ethanolic extracts from other two locations. The scavenging potential of plant extracts depends on the nature of the sample, solvents, altitude, extraction technique, and maturity of leaves. Among them, altitude is a major environmental factor that influences the composition and overall quantity of phytochemicals in plants. Plants growing at higher altitudes produce large amounts of phenolic compounds in response to environmental stresses such as low temperature and high radiation [40]; however, at lower altitudes, there is an increase in temperature which deteriorates the bioactive compounds of plants. It has also been reported that variation in lower and higher elevations significantly affects the accumulation of phytochemicals [36] which is similar to our results with relatively higher phenolics in extracts from higher altitudes than those from lower altitudes. Different studies have shown an increase in the DPPH scavenging ability of plants is directly associated with phenols and flavonoid contents [41, 42]. As most phenols are polar and contain hydroxyl groups, they are more soluble in a polar organic solvent [43]. Seo et al. [42] also reported that the phenolic content of hydroethanolic extracts of guava leaves was higher than its water extracts which is in agreement with our result with higher TPC values of ethanolic extract of guava leaves than that of other two solvents. Organic solvents like ethanol cause high penetration and denaturation of proteins which results in weakening and loosening of the cell wall structure and gives larger amounts of phenolic compounds that can contribute to the antioxidant properties [44, 45].

Plants synthesize flavonoidsfor many other purposes, and these compounds can complex with extracellular soluble proteins and with bacterial cell walls [35, 46]. MGK and MGM had significantly higher flavonoid contents than ethanol and water extracts from all locations. The solubility of flavonoids is affected due to their ability to form hydrogen bonding with solvents. Most of the polar phenolic compounds are extractable in aqueous and pure alcohol whereas non-polar flavonoids like isoflavones, flavones, and flavonols have a higher affinity toward organic solvents such as diethyl ether, dichloromethane, and ethyl acetate [47]. HPLC method was optimized for the quantitative analysis of fisetin and quercetin in water extract of guava leaves. Guava leaves constitute various secondary metabolites i.e. phenols, flavonoids, triterpenoids, glycoside, alkaloids, and saponins. The major bioactive components which show a direct correlation to antioxidant and antimicrobial activity in guava leaves are phenolic compounds. HPLC data shows that the total content of quercetin in WGK is higher than fisetin which supports the findings from Manoj et al. [48] that quercetin is a major phenolic compound in guava leaves. The activity of flavonoids is affected by both chemical properties (structure, glycosylation status, solvent system) and abiotic environmental factors (altitude, temperature, humidity, soil, pH), [49]. WGK contains a low amount of fisetin compared to quercetin but both structures of flavonoids consist of a C_2_ = C_3_ double bond and the 4-carbonyl group in ring C which plays an important role in having higher antioxidant and antibacterial properties [49, 50].

Our results show that the flavonoids such as fisetin and quercetin in WGK are higher compared to WGB and WGM, which leads us to conclude that the flavonoids content at least in fisetin and quercetin are positively correlated to the variation of altitude i.e. higher the altitude, higher the content of flavonoids and vice-versa.

MGK, EGK, and WGK had relatively higher antibacterial activity in comparison to guava leaves extracts from Bishnupur Katti and Mahajidiya This is consistent with the notion that plants growing at higher altitudes are rich source of bioactive compounds induced in response to stressful conditions [19]. Our results show both gram-positive, as well as gram-negative bacteria, were susceptible to guava leaf extracts indicating a broad spectrum of activity, which is similar to the results reported by Oncho et al., where the bacteria used for antibacterial activities were human clinical isolates [51]. However in another research finding by Biswas et al., the results were quite different where gram negative bacteria were resistant to all the solvent extracts [52]. These research findings [51, 52] show the antibacterial activity against commercially available strains and human clinical isolates, whereas our research was mainly focused on the antibacterial activity against the strains which were naturally isolated from spoiled fruits and vegetables. As mentioned above, MGK, EGK and WGK showed higher activity against bacteria isolated from different kinds of tropical and temperate fruits, however only methanol and ethanol extracts showed antifungal activity against fungal strains isolated from tomato.

The in vivo cytotoxic effects of methanol, ethanol, and water extracts of guava leaves were highly variable. Comparing LC_50_ data, potent cytotoxic activity was revealed by EGK, whereas WGK and WGM showed the least toxicity in the brine shrimp lethality assay (Table 10). This assay is considered to be a preliminary screening method for evaluating the toxicity of the extracts.

Hence, water extracts of guava leaves are nontoxic in nature and showed potential antioxidant and antibacterial activity which could be used for further applications including but not limited to the preparation of edible coating to extend the shelf-life of fruits and vegetables.

## Conclusion

In this paper, the effect of solvents (methanol, ethanol, and water) and altitude variation on phytochemicals content, antimicrobial properties, and toxicity of guava leaves was studied to explore their potential as natural preservatives. Antioxidant properties in water extract of guava leaves from Kuleshwor (WGK) exhibited no significant difference from that of other organic solvents (methanol and ethanol), suggesting the utilization of green solvents like water as an alternative for phytochemical extraction. WGK also showed similar antibacterial activity when compared with extracts from different solvents from Kuleshwor (MGK and EGK) but has higher activity when compared with extracts (WGB and WGM) from the lower altitude. This indicates that altitude plays a key role in improving the antimicrobial activity of guava leaves extracts with increasing order of concentration. Hence, our work has identified that the aqueous extract of guava leaves is rich in natural antioxidant and antimicrobial compounds which would make it ideal for application as a natural preservative.

## Supplementary Information

**Additional file 1: Table S1.** Antibacterial assay of *Psidium guajava* Linn. leaves extracts. Diameter of zone of inhibition (mm) against food spoilage bacteria isolated from spoiled fruits and vegetables. Each data represents the mean ± SD (n = 3).

## Data Availability

The authors confirm that the data supporting the findings of this study are available within the article and can be contacted to the corresponding author.

## Abbreviations

D/W: Distilled water
GAE/g dry extract: Gallic acid equivalent/gram
QE/g dry extract: Quercetin equivalent/gram
DPPH: 2,2-Diphenyl-1-picrylhydrazyl
FCR: Folin–Ciocalteu reagent
BLAST: Basic Local Alignment Search Tool
DMSO: Dimethyl sulfoxide
NaCl: Sodium chloride
LC_50_: Lethal concentration
IC_50_: Inhibitory concentration
mcg: Microgram
